# Adolescent Trials Network for HIV-AIDS Scale It Up Program: Protocol for a Rational and Overview

**DOI:** 10.2196/11204

**Published:** 2019-02-01

**Authors:** Sylvie Naar, Jeffrey T Parsons, Bonita F Stanton

**Affiliations:** 1 College of Medicine Florida State University Tallahassee, FL United States; 2 Center for HIV Educational Studies and Training Hunter College of the City University of New York New York, NY United States; 3 Health Psychology and Clinical Science Doctoral Program Graduate Center of the City University of New York New York, NY United States; 4 Hackensack Meridian School of Medicine, Seton Hall University South Orange, NJ United States

**Keywords:** Adolescent Medicine Trials Network for HIV/AIDS Interventions, implementation science, motivational interviewing, prevention cascade, youth living with HIV

## Abstract

**Background:**

The past 30 years have witnessed such significant progress in the prevention and treatment of HIV/AIDS that an AIDS-free generation and the end to the global AIDS epidemic are ambitious, but achievable, national and global goals. Despite growing optimism, globally, youth living with HIV are markedly less likely to receive antiretroviral therapy than adults (23% vs 38%). Furthermore, marked health disparities exist regarding HIV infection risk, with young men of color who have sex with men disproportionately affected. A large body of research has identified highly impactful facilitators of and barriers to behavior change. Several efficacious interventions have been created that decrease the rate of new HIV infections among youth and reduce morbidity among youth living with HIV. However, full benefits that should be possible based on the tools and interventions currently available are yet to be realized in youth, in large part, because efficacious interventions have not been implemented in real-world settings. *Scale It Up* (SIU) primarily aims to assemble research teams that will ultimately bring to practice evidence-based interventions that positively impact the youth HIV prevention and care cascades, and in turn, advance the fields of implementation science and self-management science.

**Objective:**

This paper aims to describe the structure of the U19-SIU and the effectiveness-implementation hybrid trials, as well as other center-wide protocols and initiatives, implemented within SIU.

**Methods:**

SIU will achieve its aims through 4 individual primary protocols, 2 center-wide protocols, and 3 cross-project initiatives.

**Results:**

SIU was funded by National Institute for Child Health and Human Development (U19HD089875) and began in October 2016. As of November 2018, 6 SIU protocols have launched at least the first phase of work (ATN 144 SMART: Sequential Multiple Assignment Randomized Trial; ATN 145 YMHP: Young Men’s Health Project; ATN 146 TMI: Tailored Motivational Interviewing Intervention; ATN 153 EPIS: Exploration, Preparation, Implementation, Sustainment model; ATN 154 CM: Cascade Monitoring; ATN 156 We Test: Couples' Communication and HIV Testing). Further details can be found in the individual protocol papers.

**Conclusions:**

To date, the youth HIV research portfolio has not adequately advanced the important care area of self-management. SIU protocols and initiatives address this broad issue by focusing on evaluating the effectiveness and implementation of self-management interventions. SIU is highly innovative for 5 primary reasons: (1) our research framework expands the application of “self-management”; (2) the 4 primary protocols utilize innovative hybrid designs; (3) our Analytic Core will conduct cost-effectiveness analyses of each intervention; (4) across all 4 primary protocols, our Implementation Science Core will apply implementation scales designed to assess inner and outer context factors; and (5) we shall advance understanding of the dynamics between provider and patient through analysis of recorded interactions.

**International Registered Report Identifier (IRRID):**

DERR1-10.2196/11204

## Introduction

The last 30 years have witnessed significant progress in the prevention and treatment of HIV/AIDS. Combination antiretroviral therapy (ART) has transformed HIV infection from a rapidly debilitating, fatal disease into a manageable chronic disease with high potential for a healthy life for multiple decades [1,2]. Combinations of >25 formulations of 6 classes of ART maintain the effectiveness of drug therapy in reducing viral transmission. Combined with widely available, accurate, and rapid HIV testing, pre-exposure prophylaxis (PrEP) for individuals at high risk, and universal viral suppression for those infected, an AIDS-free generation and the end to the global AIDS epidemic are ambitious, but achievable national and global goals [3,4].

However, despite growing optimism about this potentially achievable outcome, the epidemic remains a major and increasing cause of morbidity and mortality among adolescents and young adults (hereafter called “youth”) and ethnic and racial minorities. Globally, youth living with HIV (YLH) are markedly less likely to receive ART than adults (23% vs 38%) [3,5-7]. In the United States, while the overall HIV incidence from 2003 to 2014 decreased by 25%, among youth aged 13-24 years, it has increased by 43% [8]. Moreover, among youth, new infections have not been evenly distributed. Several minority groups have been overly represented; almost three-fourths of new infections were among men who have sex with men (MSM), and over half of new infections were among African American youth [9]. Young MSM of color continue to see disproportionate HIV infection rates, and these clear disparities have guided the focus for the National HIV/AIDS Strategy for the United States [10].

For ART to be effective, YLH must develop self-management behaviors at every stage of the HIV treatment cascade—linkage to care, timely initiation of care, persistence, and adherence to ART. Similarly, youth at high-risk for HIV infection must develop self-management behaviors to be fully engaged in the HIV prevention cascade—routine HIV and sexually transmitted infections testing and PrEP knowledge, access, uptake, and adherence when warranted [10-12]. While multiple barriers across range systems affect the HIV prevention and treatment cascades for youth, self-management interventions focus on how to negotiate these barriers in their current state, developing resilience even in the face of such obstacles. A quarter century of behavioral intervention research has focused on improving self-management for primary and secondary HIV prevention. This large body of research has identified highly impactful facilitators of and barriers to behavior change and has created several efficacious interventions that decrease the rate of new HIV infections among youth and reduce morbidity among YLH. However, full benefits that should be possible based on the tools and interventions currently available have yet to be realized in youth, in large part, because efficacious interventions have not been implemented in real-world settings. As a recent systematic review concluded, “As we move towards an era of universal treatment for HIV, the clinical and public health benefits of widening access to ART for adolescents will not be realized until cost-effective and sustainable service delivery interventions are widely implemented” [13].

Despite the success of the Center for Disease Control’s (CDC’s) program for disseminating evidence-based HIV-related behavioral interventions, a growing body of literature highlights substantial barriers to the effective implementation of these interventions in real-world settings [14] particularly those addressing self-management. Even less attention has been paid to the study of the implementation of behavioral interventions in HIV care settings [15], particularly in adolescent HIV clinics and community-based organizations. The *Scale It Up* (SIU) U19 was funded as a National Institutes of Health cooperative agreement as part of the Adolescent Medicine Trials Network for HIV/AIDS Interventions (ATN). SIU is committed to the development and assessment of the effectiveness of theoretically and developmentally sound interventions to improve HIV prevention and care self-management and to accelerating the pace of implementation. The primary aim of SIU is to assemble research teams that will develop, test, and bring to practice theoretically and developmentally sound self-management interventions that positively impact the youth HIV prevention and care cascades. SIU protocols focus on implementing interventions that have already been shown to be culturally appropriate and efficacious in minority populations most impacted by the epidemic. By utilizing common models and methods across protocols, we hope to advance the fields of implementation science (IS) and self-management science.

## Methods

### Aims

**Figure 1:**
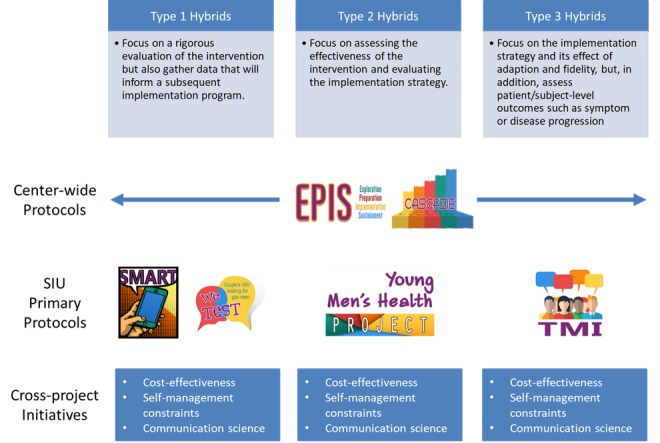
The effectiveness-implementation hybrid designs used in *Scale It Up* (SIU).

SIU will achieve its aims through 4 primary study protocols (ATN 144 SMART: Sequential Multiple Assignment Randomized Trial; ATN 145 YMHP: Young Men’s Health Project; ATN 146 TMI: Tailored Motivational Interviewing Intervention; ATN 156 We Test: Couples' Communication and HIV Testing) [16-18], 2 center-wide protocols (ATN 153 EPIS and ATN 154 Cascade Monitoring; Carcone et al, under review, and Pennar et al, under review), and 3 cross-project initiatives. A substantial amount of literature underscores the importance of shortening the time from conceptualization of a research idea to service delivery. This concern has led to the development of *effectiveness- implementation hybrid designs* to facilitate the transition of promising interventions into practice [19]. *Type 1 hybrid* designs maintain a primary focus on a rigorous evaluation of the intervention but also gather data that will inform a subsequent implementation program. *Type 2 hybrids* place a dual focus on assessing the effectiveness of the intervention and evaluating the implementation strategy. *Type 3 hybrid* designs also focus on the implementation strategy and its effect of adaption and fidelity, but, in addition, assess patient-level or subject-level outcomes such as symptoms or disease progression [19,20]. The 4 SIU primary study protocols include 2 Type 1 hybrids, 1 Type 2 hybrid, and 1 Type 3 hybrid. Two additional center-wide protocols measure contextual factors and cascade outcomes across the primary studies, and the 3 cross-project initiatives address cost-effectiveness, self-management constructs, and communication science within each protocol (Figure 1).

### Scale It Up Structure

**Figure 2:**
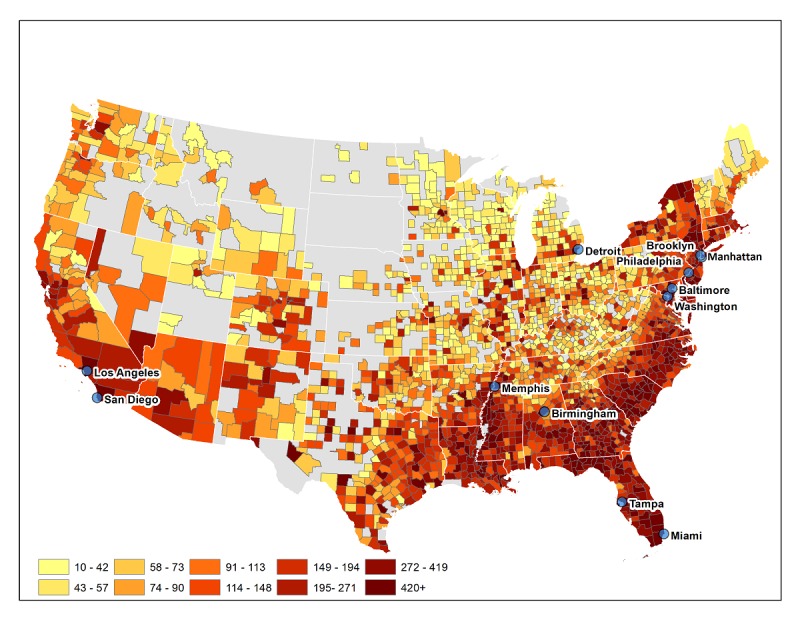
The HIV incidence in the United States and subject recruitment venue locations.

SIU is organized into 3 cores as follows: Management Core (MC); Analytic Core (AC); and an Implementation Science Core (ISC). The MC includes a Recruitment and Enrollment Center that is responsible for protocol development, project management, clinical site communication, recruitment and retention, and data collection and management. Subject recruitment venues (SRVs) are both Web-based and actual physical sites (12 sites, 10 of which provide HIV care). Our SRVs were selected on the basis of the HIV incidence (as shown in Figure 2) and based on previous successful experience with the enrollment of youth into intervention trials. Virtual recruitment strategies include the following: (1) *Social Media Recruitment* via national ad campaigns on social media sites; (2) *Geosocial Networking Apps Recruitment* —staff will also recruit through ads on geosocial networking dating apps using a pop-up message shown when a user first logs in and through a message sent by the app directly to the user’s inbox; and (3) *Text-based Recruitment—* flyers and other recruitment materials will be distributed to HIV-related organizations and those serving youth. Using Trumpia, a popular short message service (SMS) text messaging marketing service, interested youth will text a keyword (eg, “RESEARCH”) to a 5-digit number (eg, 99-000) to learn about how to screen for the protocols.

In addition, the MC includes a Community Engagement Center, with a Youth Community Advisory Board (YCAB) consisting of youth representatives from each of the 12 physical sites within SIU. The group meets virtually on a monthly basis and convenes annual in-person meetings. The SIU research team does not take the importance of meaningful community involvement in all aspects of our research lightly [21], with several of us having devoted a substantial portion of our research careers to cultivating such relationships [22-32]. Our YCAB develops their own strategic plan for community engagement, provide commentary on summaries of the protocols before they go to the field, and are included in discussions of progress and problems. The YCAB collaborates with investigators on how to best inform the communities about the planned research, that is, posting descriptions of the research projects at each site and discussions in advance of implementation with local staff and community representatives.

The YCAB will be involved in presenting the findings so that the community can understand them and in a fashion that will reach and be beneficial to community members. Furthermore, community and ATN provider input will be especially important with regard to implementing successful interventions beyond the network.

The AC provides optimal analytic support for the protocol development with the MC, statistical analysis plans for quantitative data, and the development of new methodologies and analytic strategies to accelerate the time from idea generation to program delivery. In addition, the AC facilitates the integration of scientific efforts and resources (including empirical data) across multiple research projects in a cost-effective way by providing a virtual platform for resource coordination and sharing and provide analytic support for center-wide protocols and initiatives. Finally, the AC aims to enhance the research capacity of participating institutes or investigators in areas of adolescent HIV self-management and IS through training and mentoring in advanced and innovative research methodologies, fostering high-quality research, data sharing, and improving the scholarly productivity of the network investigators, especially early career investigators.

The ISC facilitates a unified approach to IS research by applying a unified IS model to strengthen planning and implementation. The ISC provides core measures for understanding contextual factors and assessing intervention fidelity and maintains a library of categorized research papers. The ISC develops facilitator training and support resources and tools for wide-scale intervention implementation and researchers’ strategies or commercialization models for publishing the intervention products or preparing for the next steps with the interventions. Finally, the ISC develops and maintains the SIU website both internal for the center, as well as for the public, and supports early-stage investigators to develop IS studies.

### Regulatory

SIU uses a single Institutional Review Board (IRB) to accelerate the timeline of clinical research. All SRVs and investigator institutions sign reliance agreements with the IRB of record, which is located at Florida State University. While SRVs receive regular updates and notices of continuations or changes in protocol, amendments are only submitted to the IRB of record. A waiver of parental consent or assent is obtained for participants who are 15-17 years old. All clinical trials are registered on ClinicalTrials.gov, and all protocols utilize the ATN Certificate of Confidentiality. SIU utilizes a single monitoring system for all protocols to harmonize review standards across protocols. The review process of the most vulnerable protocol will be applied to all SIU protocols, thereby ensuring adequate oversight. An independent study monitoring committee consists of 3 independent experts who possess the relevant expertise (eg, HIV-related research and prevention, adolescent medicine, and sexual health) to evaluate each center protocol and who do not have a conflict of interest. The committee will review each protocol and monitor data and safety monitoring every 6 months, with additional ad-hoc reviews as necessary.

## Results

### Scale It Up Protocols and Initiatives

The SIU U19 cores evaluate and prepare for implementation self-management interventions to increase the likelihood that youth will be adherent with each step of the HIV prevention and care cascades with 4 individual effectiveness- implementation hybrid trial protocols and 2 center-wide protocols that assess contextual implementation factors and cascade outcomes. Furthermore, the cross-project initiatives of cost-effectiveness analyses, analysis of a theoretically driven self-management model, and analysis of patient-provider communication are incorporated into multiple protocols.

### Effectiveness-Implementation Hybrid Trials

**Figure 3:**
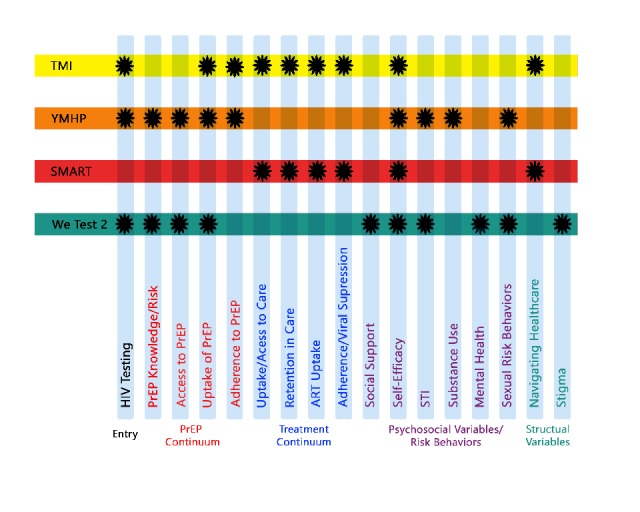
Scale It Up (SIU) overall. PreP: pre-exposure prophylaxis. STI: sexually transmitted infection.

The individual project protocols are described later in this supplement. The following is a brief synopsis of each (Figure 3).

*ATN 144* is a SMART design that tests the sequencing of SMS text message cell phone support (CPS), and contingency management in youth nonadherent to ART [16]. All study procedures will be conducted over the Web or by phone so that youth need not attend a clinic to participate, and clinics only need to refer participants to the study website instead of staffing for recruitment. As a Type 1 effectiveness-implementation hybrid, while implementation context will be assessed, the focus is on the intervention effectiveness in true real-world settings (in and out of the clinic and across the nation). Youth are first randomized to 12 weeks of CPS versus SMS text messaging. Several critical issues surrounding incentives and intervention tapering are explored through a second randomization—(1) if CPS or SMS text messaging for 12 weeks is successful, does viral load (VL) suppression persist longer if the intervention dose is tapered (ie, less frequent) over the next 12 weeks versus terminating CPS and receiving standard care; (2) if CPS or SMS text messaging is not successful in the first 12 weeks, will VL suppression occur if incentives for intervention adherence are added for 12 more weeks; and (3) if CPS or SMS text messaging is not successful, will VL suppression occur if youth receive the other intervention condition (with the addition of incentives) or if they are allowed 12 more weeks within their initial condition (with the addition of incentives). Details are provided in ATN 144 SMART [16].

*ATN* *145,* the YMHP, tests a 4-session intervention integrating motivational interviewing (MI), personalized feedback, and problem-solving skills to reduce condomless anal sex (CAS) and substance use among HIV-negative young MSM (Parsons et al, under review). Previous studies have found that youth receiving YMHP reported markedly greater reductions in CAS and substance use than youth in the comparison condition, and the CDC recently rated YMHP as “Best Evidence” and included it in the compendium of Evidence-Based Interventions for HIV Prevention [33]. YMHP now requires evaluation through an effectiveness-implementation Hybrid Type 2 trial to both provide the best evidence to inform practitioners as to which approach to delivery (clinic-based or phone-based) is most effective and cost-effective prior to dissemination on a wider scale (comparative effectiveness design) while simultaneously testing a model of intervention implementation through training local supervisors and studying contextual barriers and facilitators. The trial will assess HIV prevention-focused outcomes (sexually transmitted infections, PrEP uptake) and self-management behaviors (condom use, reduction in substance use, PrEP adherence if uptake is achieved), as well as intervention fidelity. Participants complete the 4-session intervention and an immediate posttest assessment 3 months after the baseline. They are then assessed every 3 months for 12 months postintervention. Details are provided in ATN 145 YMHP (Parsons et al, under review).

*ATN 146*, TMI [17] addresses the training of health care providers to deliver MI, a method of communication shown to improve multiple points across the youth prevention and care cascades [27,34-45]. Pilot work to develop the TMI included tailoring initial workshop training based on innovative methods in communication science, developing efficient fidelity measurement, and preliminary testing of implementation strategies. The effect of TMI on fidelity to the original evidence-based program, and secondarily on cascade-related outcomes (see ATN 154 below), will be achieved by using a dynamic waitlist-controlled design with 150 providers nested within the 10 clinical care sites of our 12 physical SRVs, yielding 5 clusters to receive TMI. For each randomization, 2 of the clinics receive TMI, and the others remain in the wait-list condition. This will continue until the fifth cluster has been randomized to TMI. After 1 year of TMI’s external facilitation based on the dynamic adaptation process, second randomization will compare internal facilitator monitoring and coaching plus the encouragement of communities of practice to communities of practice alone. Fidelity will be assessed using ratings of standard patient interactions on a quarterly basis through the 24 months of intervention and an additional 6 months of follow-up. As a Hybrid Type 3 Implementation-Effectiveness, the primary focus is also on exploring the role of the barriers and facilitators to implementation with repeated qualitative interviews and quantitative surveys of implementation context (see ATN 153 below). Details are provided in ATN 146 TMI [17].

*ATN 156,* Enhancing Sexual Safety: Couples’ Communication and HIV Testing Among YMSM (We Test), is a comparative effectiveness trial of couples HIV testing and counseling (CHTC) for adolescent age (15-19 years) same-sex male couples [18]. This design tests the added benefits of adjunct intervention components delivered prior to receipt of CHTC-Assertive Communication Training (CT) videos viewed by the couple together and individually delivered MI-based Communication Skills Training (MI-CST). These target the development of communication skills necessary to participate fully in HIV prevention and sexual safety discussions inherent to CHTC. This protocol will assess a continuum of intervention packages to address the developmental needs of young MSM (CHTC; CT videos + CHTC; MI-CST + CT videos + CHTC) to identify which package optimizes outcomes while minimizing delivery cost. Participants complete the intervention session and an immediate posttest assessment 3 months after the baseline; they are then assessed every 3 months for 9 months postintervention. Details are provided in ATN 156 We Test [18].

### Center-Wide Protocols and Initiatives

The 4 primary protocols, with support from the 3 cores, *de* *facto* form center-wide protocols and cross-project initiatives, exploiting synergies created by our U19 and creating value-added scientific contributions that would not be possible from individual projects alone or through traditional R01-level funding. First, individually and collectively, the 4 research protocols are guided by the same IS conceptual model. SIU employs the National Institutes of Health’s definition of implementation “the use of strategies to introduce or change evidence-based health interventions within specific settings” [46]. While implementation and dissemination models now abound, we selected an adapted version of Aaron’s EPIS model to guide our work as it is logical, evidence-based [47,48], supported by a growing number of evidence-based instruments [49], and has broad reach among youth HIV researchers (see Figure 1). Thus, ATN 153 EPIS (Carcone et al, under review), described later in this supplement, is a mixed-methods study that includes qualitative interviews and quantitative surveys with staff at 12 physical SRVs to assess barriers and facilitators of the adoption and use of evidence-based behavioral interventions in general and project-specific interventions. Anticipated factors are assessed at the baseline, factors identified during implementation are assessed at 12-18 months postbaseline, and factors identified during sustainment are assessed at 24-30 months postbaseline.

The 10 clinical care sites in SIU will provide deidentified data from electronic health records regarding demographics and HIV treatment cascade variables annually in ATN 154 Cascade Monitoring (Pennar et al, under review). These data will not only serve as outcomes of implementation but also will provide rough but rapid estimations of demographic indices and measures of response (such as new care entry and percent viral suppression) that can provide useful indicators of the epidemic at large for ATN strategic planning. In addition, we hoped to capture prevention cascade variables, but early interviews with the clinical sites determined that consistency in electronic record documentation of these variables was not sufficient for this protocol. Thus, the ATN is developing a new cross-network protocol to assess the capability of all recruitment venues to provide consistent and valid prevention and treatment cascade records and develop an intervention to achieve this end (ATN 162).

Several initiatives are represented across the protocols. First, the 3 primary protocols gathering data from youth (ATN 144 SMART, ATN 145 YMHP, and ATN 156 We Test) are guided by the same theoretical model for self-management [50]. According to the Five Components Model, self-management includes 5 essential skills—*problem solving*, *decision making*, *resource utilization*, *forming of a patient/heath care provider partnership*, and *taking action* [50,51]. Thus, all 3 protocols will include the following measures: (1) the BRIEF [52] to assess both problem solving and decision making; (2) Patient Activation Measure [53] in which participants rate the relationship with their providers and the degree to which they are involved in their care using a 4-point Likert scale; (3) an adapted version of the 12-item Services and Support measure utilized in ATN 004 (Healthy Choices) [54] to capture participant self-report of health care and related services (eg, emergency department care, hospitalizations, residential substance use treatment facilities, case management, and support group; *resource utilization*); and (4) self-reported medication adherence and condom use and viral suppression (*taking action*). The model will be tested utilizing Structural Equation Modeling with 820 youth.

**Figure 4:**
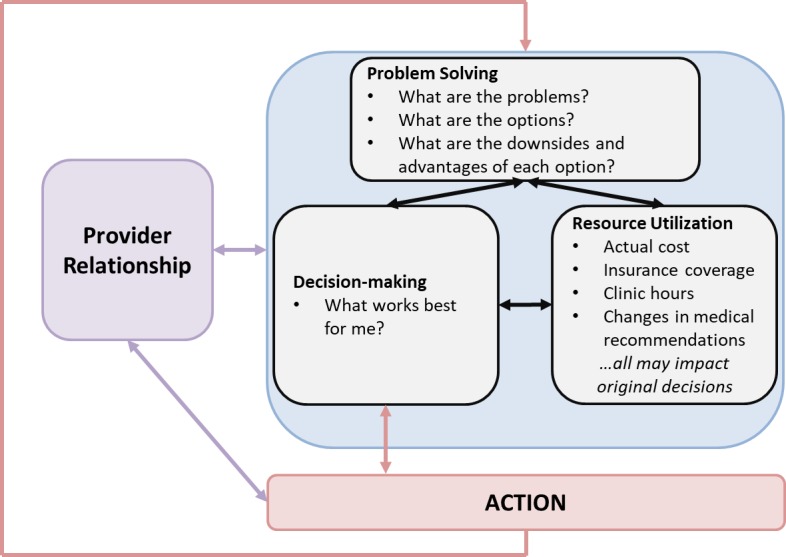
Self-management components.

Second, given the importance of *provider-patient interactions* in encouraging youth self-management, we plan to identify provider communication behaviors that predict self-management and health outcomes using innovative sequential analysis [55,56] of coded audiorecordings across all 4 primary protocols. We will use MI as a framework (eg, emphasizing autonomy and reflections of change talk) using the Minority Youth Sequential Code of Process Exchanges (MY-SCOPE), adapted from existing MI coding schemes [57]. This analysis generates transition probabilities for patient-provider communication sequences, allowing provider communication behaviors to predict subsequent youth communication and, then, later link to youth outcomes (Figure 4).

Finally, we will determine the relative cost-effectiveness of interventions within each primary protocol to assist in the implementation and rapid diffusion of effective and cost-effective interventions in the practice community. Each protocol will collect detailed resource use for the interventions using consistent methods. These data will be assigned standard cost weights developed by the AC for performing incremental cost-effectiveness analyses across studies. The use of resource use measures with standard cost values will assure that the economic analyses associated with the clinical trial meet the International Society for Pharmacoeconomics and Outcomes Research Good Practices for Economic Evaluation Alongside Clinical Trials [58]. We will use a modification of The Drug Abuse Treatment Cost Analysis Program (DATCAP) [59,60], combined with study contract and expenditure records, capturing both contractually allowed expenditures, and relevant expenditures supported from other budgets, to estimate the cost of the CPS and SMS text messaging conditions.

The DATCAP is a standardized instrument that estimates the economic cost of alcohol and other drug treatment programs (eg, personnel, facilities, and supplies). Client case flow data are incorporated to determine the average weekly and annual cost or client for each service type, average cost per intervention episode, and marginal cost per contact.

When available, data on sexual activity will be collected to be used with recent national estimates [61] of cost per HIV infection avoided, and/or on cost per HIV infection delayed, a more conservative measure. This will allow the estimation of potential cost savings in a reduction in new HIV infections. If warranted by study findings, we will use a previously validated Markov decision analysis model [62] to estimate the expected treatment cost savings resulting from the increase in time with suppressed VL. The resource use measures and cost data collected will be used to develop budget impact scenarios to help inform “scale up” and diffusion planning for the most cost-effective intervention combinations.

## Discussion

### Principal Findings

A dramatic decline in HIV transmission is achievable with currently available protocols and interventions (including ART, PrEP, rapid and widespread testing); however, such decline has not yet been realized among youth. Our goal must be complete self-management as mathematical modeling indicates that even achieving 90% compliance at different points on the cascade is insufficient to curb the epidemic [11]. Self-management is critical and complex at any age, but may be especially challenging among adolescents and emerging adults as they transition from a largely dependent to a more independent status (“transition to self-management”) [63] during a developmental period marked by identity exploration, development of new social networks, increased opportunities and choices, both positive and risk-laden [64], and increased independence and risk-taking behavior [65,66]. The transition to adult health care can be abrupt and can occur with little preparation because age, rather than developmental maturity, triggers the transition. Given these developmental and systemic challenges, it is not surprising that self-management tends to deteriorate during this transitional period [54,67]. New approaches to HIV education, prevention, and treatment of youth must be integrated with issues in self-management to achieve an AIDS-free generation and/or the end of the AIDS epidemic by 2030 in the United States and globally [3,4]. To date, the youth HIV research portfolio has not adequately advanced this important care area [13]. SIU protocols and initiatives address the broad issue of persistent disproportionately high rates of HIV infection among youth and failure to engage in the HIV prevention and treatment cascades [68] by focusing on evaluating the effectiveness and implementation of self-management interventions.

SIU is highly innovative for 5 primary reasons. First, the 4 primary study protocols utilize innovative hybrid designs to capture critical information to facilitate and expedite the introduction of effective programs into practice by exploring important implementation issues while testing the effectiveness [19,20]. In keeping with the theme of expeditiously and parsimoniously assessing intervention effectiveness and moving it to practice, we shall use innovations in evaluating the intervention effectiveness, a SMART [69], 2 comparative effectiveness trials [70], and a Dynamic Wait List-controlled trial [71]. Second, our research framework expands the application of “self-management” from the *management of chronic disease* [72,73] to the *prevention* [74] *and management of chronic disease*, HIV/AIDS. We will test an innovative theoretical model of self-management over time among all enrolled youth in SIU protocols (N=500) using standardized measures. Third, our AC will conduct cost-effectiveness analyses of each intervention within the 4 primary study protocols, enabling us to compare the effectiveness and cost-effectiveness to further shorten the research to practice gap. Fourth, across all 4 primary protocols, our ISC will apply implementation scales designed to assess *inner* and *outer context* factors based on a strong theoretical model [47-49,75] to determine their relevance to fidelity and sustainability both for evidence-based behavioral practice in general and for interventions grounded in MI as the method of communication. Fifth, we shall advance understanding of the dynamics between the provider and patient by introducing an analytic approach from Communication Science (“sequential analysis”) to the provider-youth interaction across all 4 primary protocols through analysis of recorded interactions [76].

### Limitations

Challenges experienced to date include the inexperience of SRVs in single Internal Review Board and reliance agreements, recruitment and retention difficulties when staff are participants in implementation trials, recruitment of high-risk YLH who are nonadherent to ARVs outside of the clinic setting and obtaining VL data for those youth who are not regularly attending clinic, inexperience of clinical sites in electronic health record downloads, and the need for communication systems for complex protocols with multiple moving parts.

### Conclusions

In summary, the 4 SIU primary protocols, 2 center-wide protocols, 3 cross-project initiatives, and 3 supporting cores are highly integrated and carefully constructed to support the overarching themes of improved self-management on the part of YLH and at-risk youth and expeditious, but appropriate, implementation of effective prevention and treatment programs into practice in a cost-effective manner. We aspire to address specific research hypotheses concerning the HIV prevention or care cascades for youth while advancing self-management theory and IS. This agenda is dependent on the synergy of connections between these protocols, initiatives and cores and our clinical and community partners, without which these advances would not be achievable.

Given that SIU implements multiple protocols and initiatives, managing this multifaceted U19 project has proved challenging. The challenges range from recruiting participants from clinics with a smaller than expected pool to select from (both among staff and YLH) to grant transfer delays between institutions, as well as IRB and reliance agreements, which, in turn, creates a substantial bureaucratic burden. Nevertheless, these challenges did inform the cores on how to learn from and adapt to the always changing landscape of SIU. At the bureaucratic level, the cores found that maintaining close contact with IRB and financial administrators allows SIU to operate efficiently. At the clinic level, the cores learned that sites rely on ATN support for infrastructure and that even the simplest of protocols can prove difficult to implement. For example, the small pool of participants and participation from multiple sites required innovative approaches for recruitment, data collection, and interventions. As mentioned before, SIU specifically focuses on the process of improving self-management among youth (both YLH and at-risk youth). For these evidence-based practices within SIU to be effective, youth must be fully engaged in interventions at every stage of the HIV care (treatment and prevention) cascades. SIU initially seeks to implement within clinics, outside of the research context, and ultimately assess if clinics have the capacity to implement these interventions independently, thus improving health outcomes for the targeted youth populations.

## Abbreviations

AC: Analytic Core
CT: Assertive Communication Training
ART: antiretroviral therapy
ATN: Adolescent Medicine Trials Network for HIV/AIDS Interventions
CHTC: couples HIV testing and counseling
CPS: cell phone support
DATCAP: Drug Abuse Treatment Cost Analysis Program
EPIS: Exploration, Preparation, Implementation, Sustainment model
IS: implementation science
ISC: Implementation Science Core
MC: Management Core
MI: motivational interviewing
MSM: men who have sex with men
PrEP: pre-exposure prophylaxis
SIU: Scale It Up
SMART: Sequential Multiple Assignment Randomized Trial
SMS: short message service
SRV: subject recruitment venue
TMI: Tailored Motivational Interviewing Intervention
VL: viral load
YCAB: Youth Community Advisory Board
YLH: youth living with HIV
YMHP: Young Men’s Health Project
